# Evaluation of the ear ossicles with photon-counting detector CT

**DOI:** 10.1007/s11604-023-01485-0

**Published:** 2023-08-27

**Authors:** Yuka Takahashi, Fumiyo Higaki, Akiko Sugaya, Yudai Asano, Katsuhide Kojima, Yusuke Morimitsu, Noriaki Akagi, Toshihide Itoh, Yusuke Matsui, Takao Hiraki

**Affiliations:** 1 Department of Radiology, Okayama University Hospital, 2-5-1 Shikata-Cho, Kitaku, Okayama, 700-8558 Japan; 2 Department of Otolaryngology-Head and Neck Surgery, Okayama University Hospital, Okayama, Japan; 3 Department of Radiological Technology, Okayama University Hospital, Okayama, Japan; 4 Department of CT-Research and Collaboration, Siemens Healthineers, Tokyo, Japan; 5 Department of Radiology, Okayama University Faculty of Medicine, Dentistry, and Pharmaceutical Sciences, Okayama, Japan

**Keywords:** Photon-counting detector computed tomography, Energy-integrating detectors, Ear ossicles, High-resolution imaging, 3D

## Abstract

Recently, computed tomography with photon-counting detector (PCD-CT) has been developed to enable high-resolution imaging at a lower radiation dose. PCD-CT employs a photon-counting detector that can measure the number of incident X-ray photons and their energy. The newly released PCD-CT (NAEOTOM Alpha, Siemens Healthineers, Forchheim, Germany) has been in clinical use at our institution since December 2022. The PCD-CT offers several advantages over current state-of-the-art energy-integrating detector CT (EID-CT). The PCD-CT does not require septa to create a detector channel, while EID-CT does. Therefore, downsizing the anode to achieve higher resolution does not affect the dose efficiency of the PCD-CT. CT is an indispensable modality for evaluating ear ossicles. The ear ossicles and joints are clearly depicted by PCD-CT. In particular, the anterior and posterior legs of the stapes, which are sometimes unclear on conventional CT scans, can be clearly visualized. We present cases of congenital anomalies of the ossicular chain, ossicular chain dislocation, tympanosclerosis, and cholesteatoma in which PCD-CT was useful. This short article reports the usefulness of PCD-CT in the 3D visualization of the ear ossicles.

## Introduction

X-ray computed tomography (CT) is an indispensable modality for evaluating ear ossicles. CT with a photon-counting detector (PCD-CT) was recently developed to enable high-resolution imaging at lower radiation doses. The usefulness of this tool in evaluating the ear ossicles has been reported in studies in which investigational PCD-CT [1] and cadavers [2] were used. The newly released PCD-CT system (NAEOTOM Alpha; Siemens Healthineers, Forchheim, Germany) has been used clinically at our institution since December 2022. This short article further reports the usefulness of PCD-CT in the 3D visualization of the ear ossicles.

PCD-CT employs a photon-counting detector (PCD) that measures the number of incident X-ray photons and their energy.

**Fig. 1:**
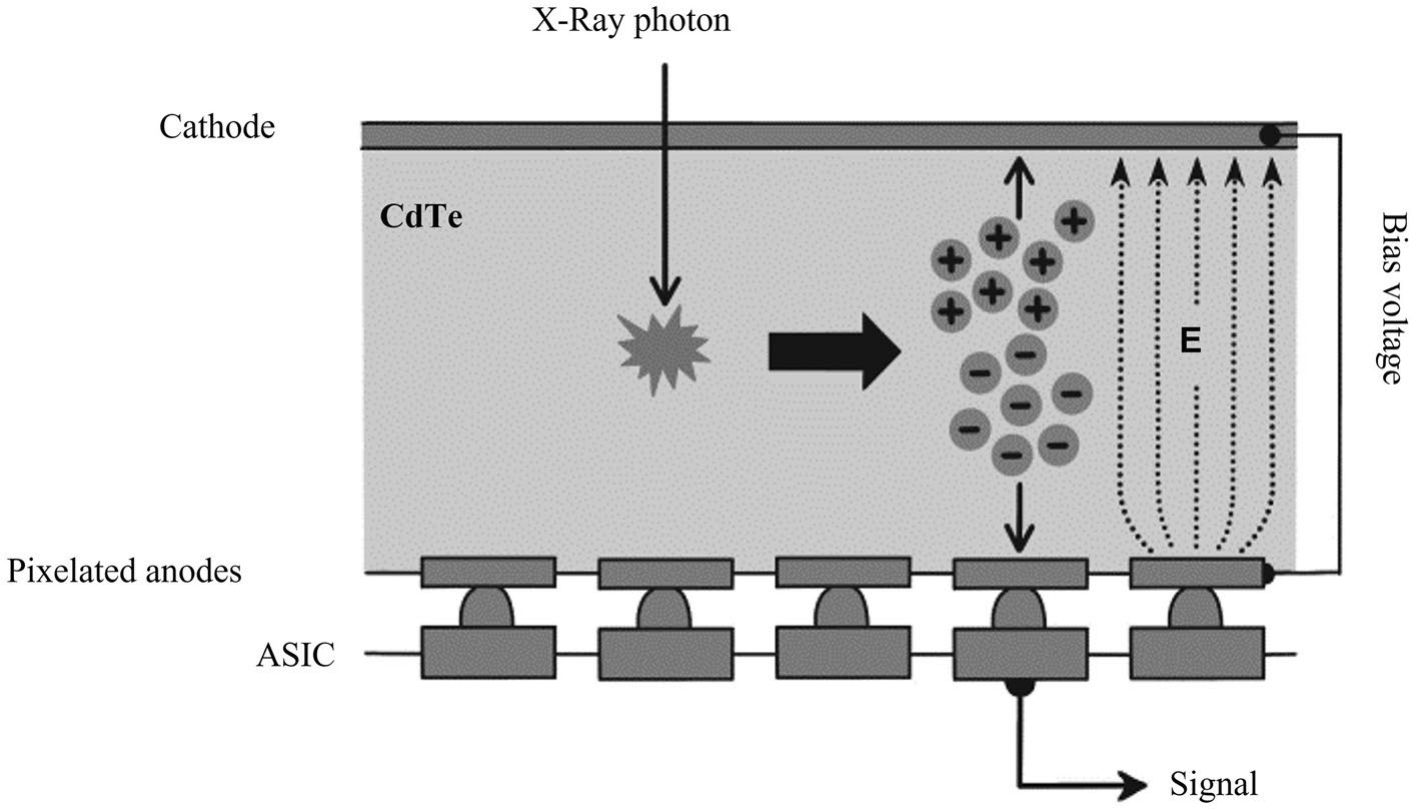
Schematic of the direct-converting photon-counting detector. The X-ray absorbed in a semiconductor—a single crystal of cadmium–telluride (CdTe)—produces electron–hole pairs that are separated by a strong electric field E between the cathode and pixelated anodes. No septa were observed between the anodes. *ASIC* application-specific integrated circuit

**Fig. 2:**
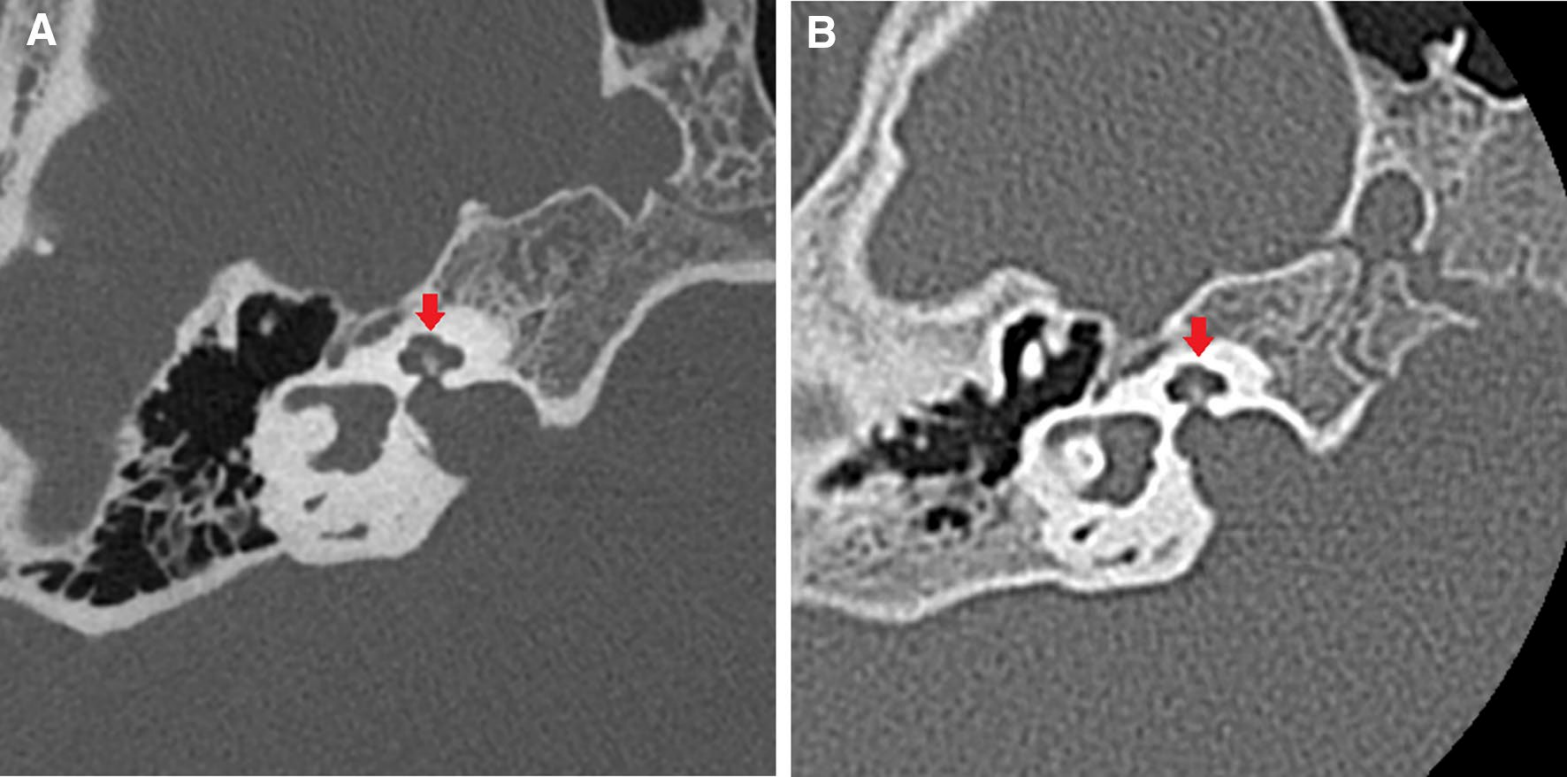
A case of a boy imaged with both EID-CT and PCD-CT for follow-up in his childhood and teenage following hearing loss. **A** PCD-CT **B** EID-CT. The internal structures of the cochlea are more clearly delineated by PCD-CT (**A**, **B**, arrows)

PCD-CT offers several advantages over the current state-of-the-art CTs that use energy-integrating detectors (EID). The PCD-CT does not require septa to create a detector channel for the EID; what serves the same purpose as a detector channel in EID are the smaller pixelated anodes (0.2 mm × 0.2 mm) that are placed on the bottom of a single crystal of cadmium–telluride (Fig. 1). Therefore, the dose efficiency of the PCD does not decrease with smaller anodes to achieve a higher spatial resolution. Compared with EID-CT with the same detector cell size (0.25 × 0.25 mm), PCD-CT shows a 19% reduction in image noise [3] (Fig. 2).

PCD is also unique in that it converts incident X-ray photons into pulses, the heights of which reflect the energy value. The energy of each X-ray photon was measured using an energy discriminator with a preset PCD energy threshold. The PCD was equipped with multiple energy discriminators allowing simultaneous energy measurements. The lowest energy threshold can eliminate electrical noise in the measurement signal, which reduces the signal-to-noise ratio in high-resolution and low-dose imaging. This mechanism reduces image noise by approximately 46% [4].

### Acquisition and imaging

The scanning and imaging parameter settings used for this study are shown in Table 1. The automatic tube current modulation was used for patient dose optimization. Filtered back-projection and iterative reconstruction techniques are available for PCD-CT image reconstruction.

**Table 1:** Acquisition and imaging parameter setting

Tube voltage	120 kV
Tube current	Quality ref. mAs 252
CTDI vol	39.9–51.6 mGy (16 cm)
Collimation	120 × 0.2 mm—UHR mode
Rotation time	0.5 s
Pitch	0.55
Kernel	Hr76
Strength of iterative reconstruction	QIR 1
Image matrix	512
Slice thickness	0.2 mm

### Demonstration of the normal ear ossicles

**Fig. 3:**
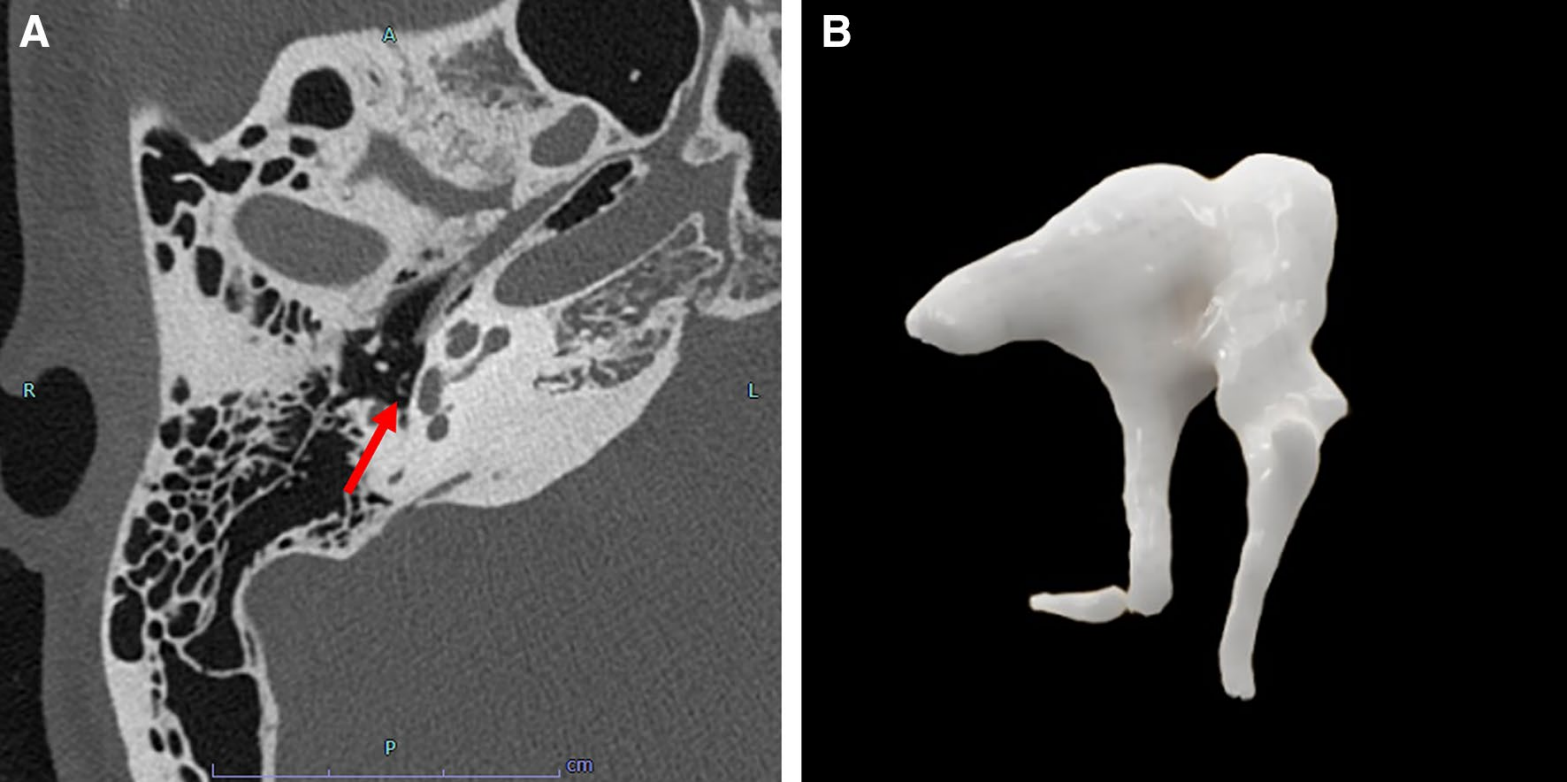
The normal ear ossicles and joints. **A** axial image **B** 3D image. The stapes is clearly demonstrated. (**A**, arrow)

The ear ossicles comprise three bones: the malleus, incus, and stapes, joined by the incudomalleolar and incudostapedial joints. The ear ossicles and joints are depicted clearly by PCD-CT (Fig. 3). In particular, the anterior and posterior legs of the stapes, which are sometimes unclear on conventional CT scans, can be clearly visualized.

### Congenital anomalies of the ossicular chain

**Fig. 4:**
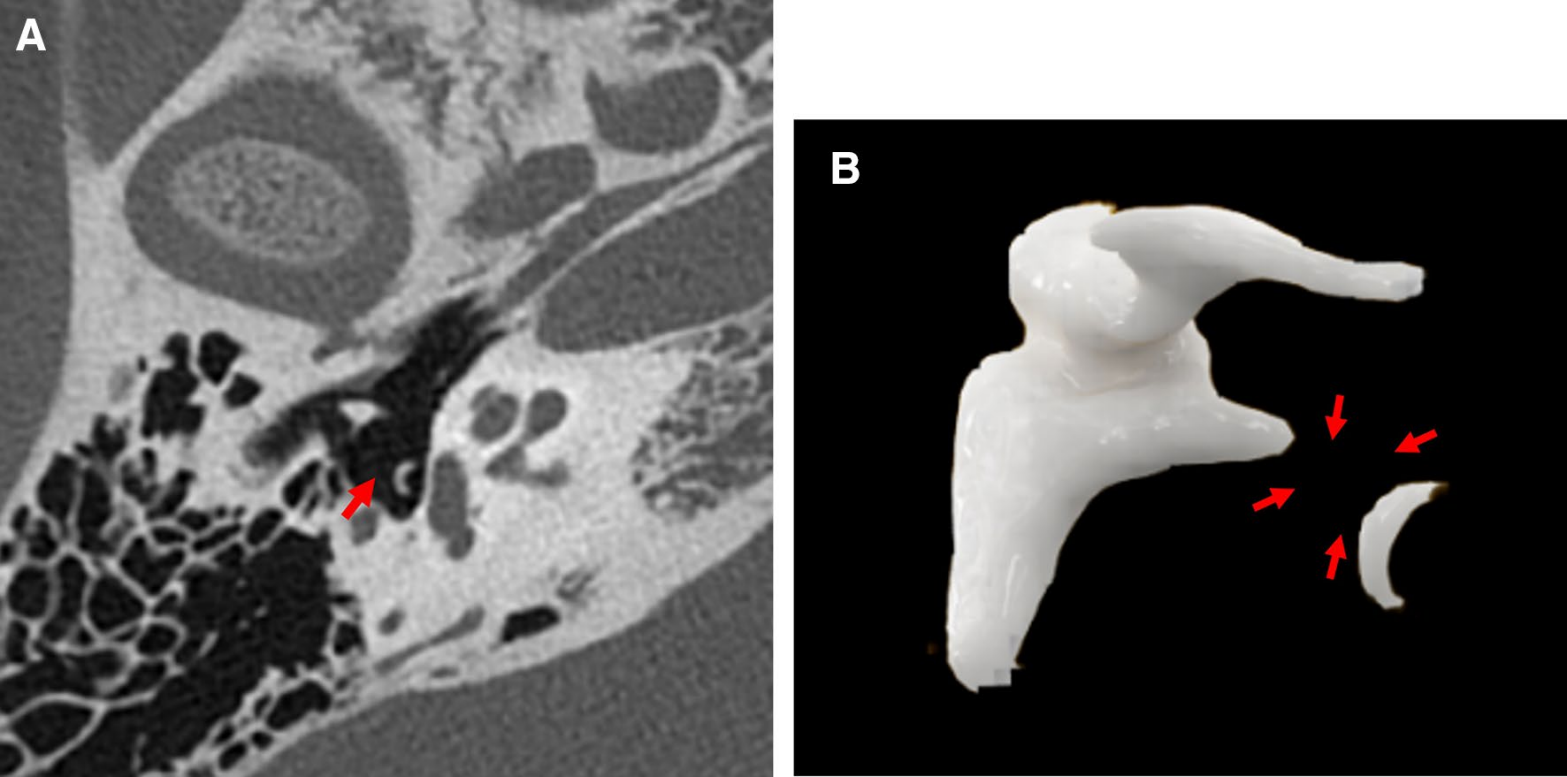
A case of congenital anomalies of the ossicular chain in a girl in her teens with a chief complaint of right hearing loss. **A** axial image **B**; 3D image. Disconnection of the incudostapedial joint is seen (**A**, **B**, arrows)

Congenital middle ear anomalies can be classified into four categories according to the Cremers and Teunissen classification [5]. We present a case of category 3, "Ossicular chain anomaly, mobile stapes footplate," which is the most frequent one (Fig. 4).

### Ossicular chain dislocation

Ossicular chain dislocation is relatively rare and may be associated with trauma to the temporal bone, although its exact incidence is unknown [6].

It is estimated that up to 50% of temporal bone fractures result in damage to the ear ossicles [7, 8]. However, ossicular chain dislocations can occur even without temporal bone fractures [9].

**Fig. 5:**
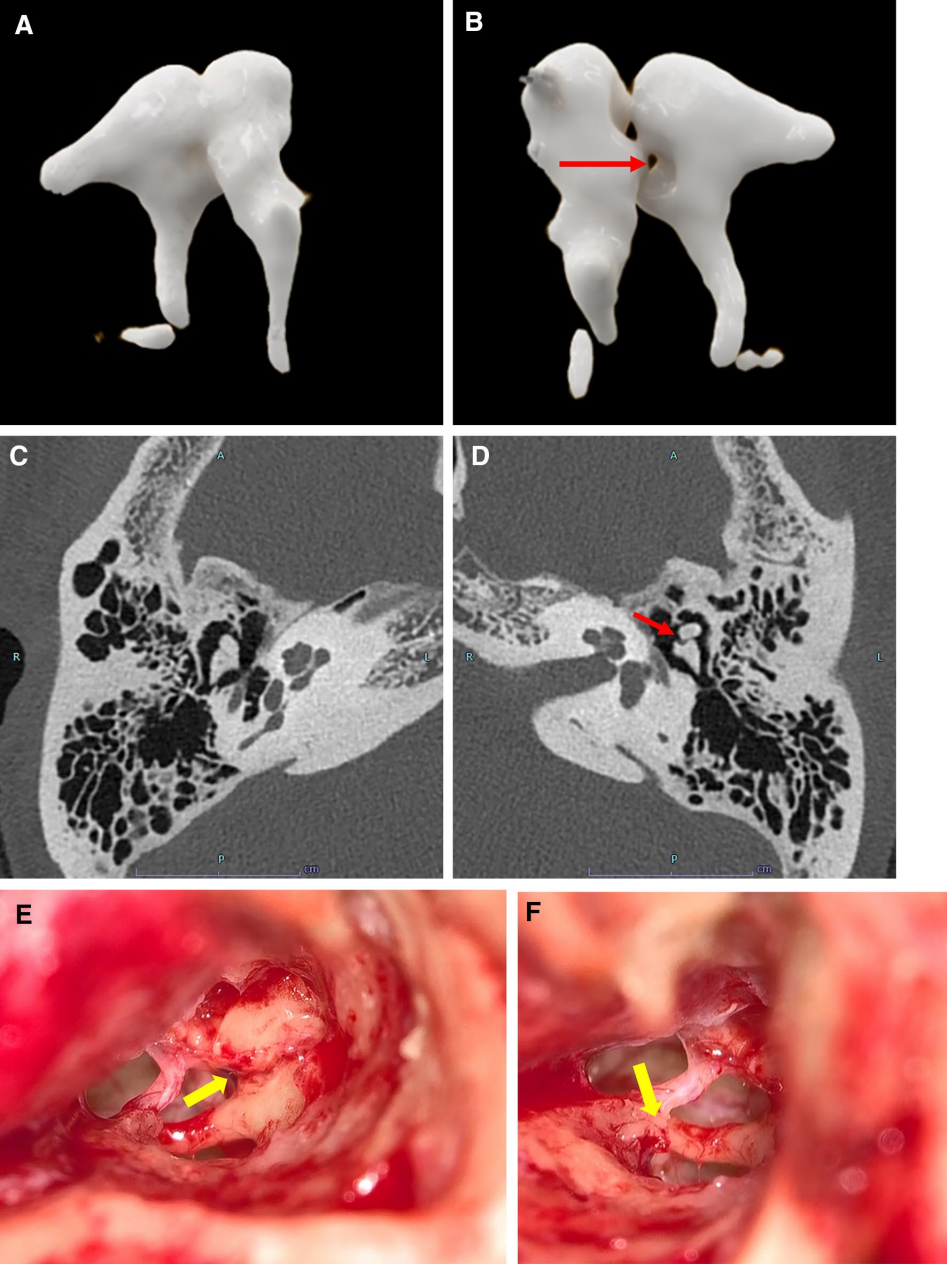
A case of traumatic ossicular chain dislocation in a woman in her 50 s with a chief complaint of left hearing loss. **A**; 3D image of the normal right ossicles. **B**; 3D image of left ossicular chain dislocation. **C**; Axial image of the normal right ossicle. **D**; Axial image of left ossicular chain dislocation. **E**, **F**; intraoperative images. PCD-CT showing dislocation of the left incudomalleolar joint in the 3D image (**B**, arrow) and axial image (**D**, arrow). The head of malleus is anteriorly dislocated in the left tympanum, unlike the normal incudomalleolar joint in the right (**A**, **C**). During the surgery, anterior deviation of the malleus was observed. Dislocation of the incudomalleolar joint (**E**, arrow). There was no evidence of incudostapedial joint disarticulation (F, arrow). Preoperative CT findings were consistent with intraoperative findings.

Radiologically, it is usually challenging to find an incudomalleolar joint dislocation [10]. However, PCD-CT can identify minute dislocations on high-resolution images. Furthermore, 3D reconstruction using PCD-CT allowed us to examine the incudomalleolar and incudostapedial joints in multiple planes and angles, thereby increasing the possibility of identifying traumatic injury structures in the temporal bone (Fig. 5).

### Tympanosclerosis

**Fig. 6:**
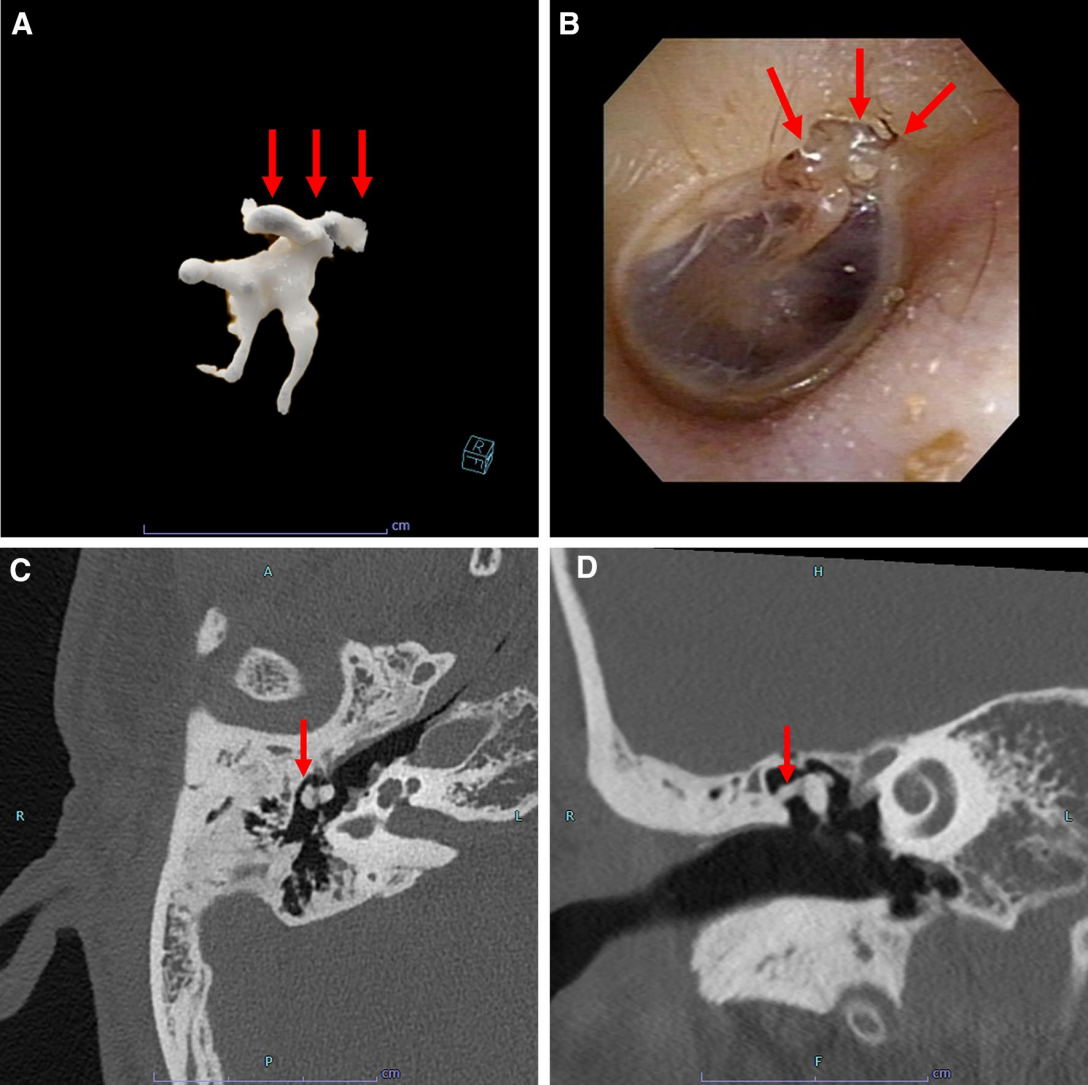
Tympanosclerosis in a man in his 40 s without related symptoms. A, 3D image, B; tympanic image, C; axial image, D; coronal image. An irregular high-density area is observed (A, C, D, arrow). This is consistent with tympanic findings (B, arrow).

Tympanosclerosis was observed as a high-density area on the CT scans (Fig. 6). Chronic suppurative otitis media is the most common etiological factor in tympanosclerosis. Tympanosclerosis of the middle ear cavity is most often observed around the malleus handle [11].

### Cholesteatoma

**Fig. 7:**
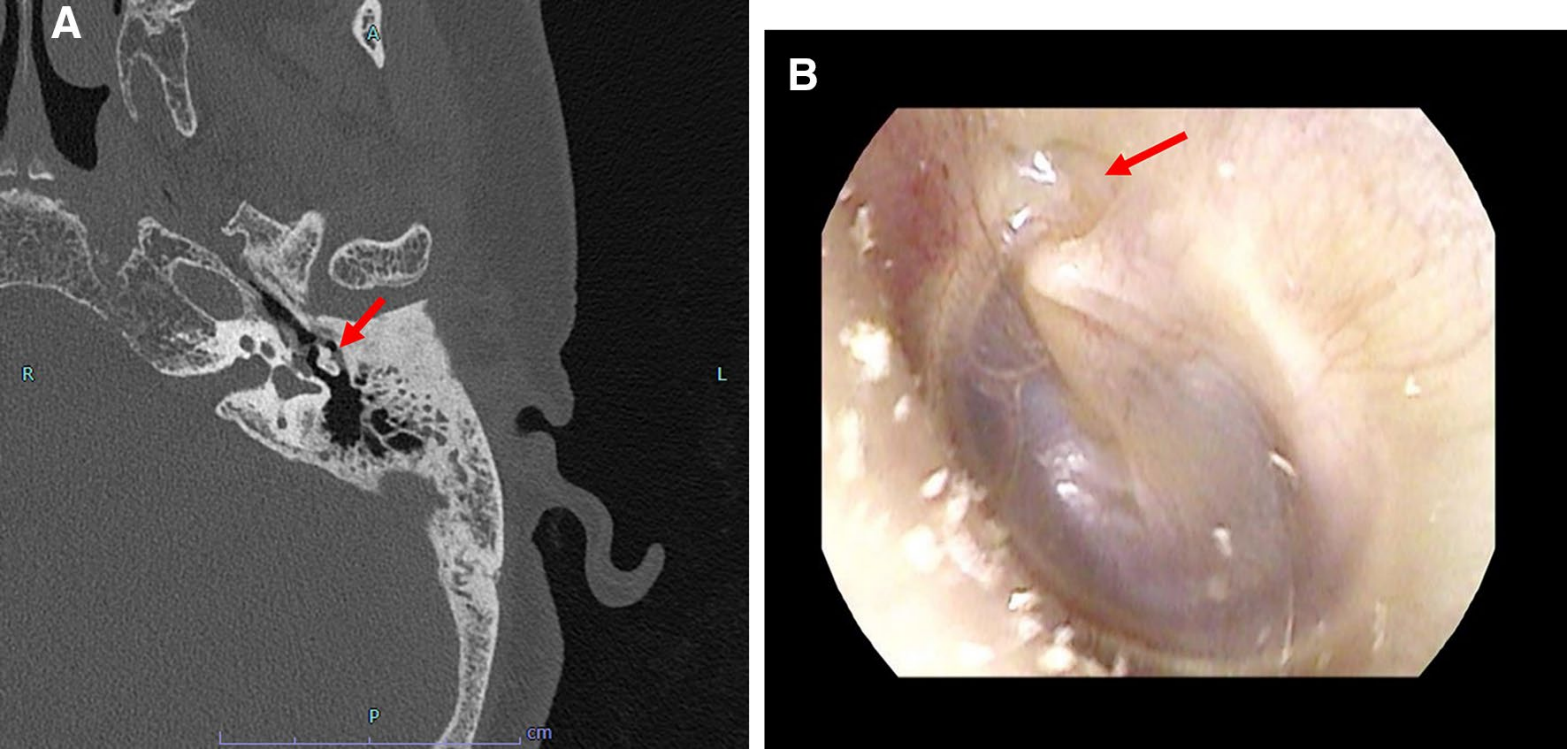
Cholesteatoma in a woman in her 60 s with a chief complaint of left hearing loss. A, axial image, B; tympanic image. The soft tissue density area is seen around the ear ossicles (A, arrow). The ear ossicles are not destroyed.　Tympanic membrane image showing consistent retraction (B, arrow).

Bone destruction is a feature of cholesteatoma, and the coexistence of a soft tissue-dense lesion in the tympanum and destruction of the ear ossicles are specific findings of cholesteatoma (Fig. 7).

## Conclusion

The ear ossicles could be clearly visualized using PCD-CT, particularly in 3D images.
